# Effects of the Rho-Kinase Inhibitor Y-27632 on Extraocular Muscle Surgery in Rabbits

**DOI:** 10.1155/2017/8653130

**Published:** 2017-07-26

**Authors:** Ji‐Sun Moon, Hyun Kyung Kim, Sun Young Shin

**Affiliations:** ^1^Department of Ophthalmology and Visual Science, Seoul St. Mary's Hospital, College of Medicine, The Catholic University of Korea, No. 222 Banpo-daero, Seocho-gu, Seoul 06591, Republic of Korea; ^2^Department of Ophthalmology, St. Vincent's Hospital, College of Medicine, The Catholic University of Korea, No. 93 Jungbu-daero, Paldal-gu, Suwon 16247, Republic of Korea

## Abstract

**Purpose:**

To evaluate the effect of the Rho-kinase inhibitor Y-27632 on postoperative inflammation and adhesion following extraocular muscle surgery in rabbits.

**Methods:**

The superior rectus muscle reinsertion was performed on both eyes of 8 New Zealand white rabbits. After reinsertion, the rabbits received subconjunctival injections of the Rho-kinase inhibitor and saline on each eye. To assess acute and late inflammatory changes, Ki-67, CD11*β*+, and F4/80 were evaluated and the sites of muscle reattachment were evaluated for a postoperative adhesion score and histopathologically for collagen formation.

**Results:**

F4/80 antibody expression was significantly different in the Rho-kinase inhibitor-injected group at both postoperative day 3 and week 4 (*p* = 0.038, 0.031). However, Ki-67 and CD11*β*+ were not different the between two groups. The difference in the SRM/conjunctiva adhesion score between the two groups was also significant (*p* = 0.034)*. Conclusion*. Intraoperative subconjunctival injection of the Rho-kinase inhibitor may be effective for adjunctive management of inflammation and fibrosis in rabbit eyes following extraocular muscle surgery.

## 1. Introduction

During the surgical wound healing after extraocular muscle surgery, the damaged tissue must restore its original function and structural integrity. Therefore, it is important to prevent inflammation and fibrosis, as the perfect restoration of previous tissue architecture without scar is the purpose of wound healing. The major complications of strabismus surgery are postoperative adhesions and fibrosis, which can cause limitation of ocular movement and adversely affect the surgical outcome. These adhesions can occur with various tissues, extraocular muscles, sclera, orbital fat, intermuscular membrane, Tenon's capsule, and conjunctiva.

Numerous studies by strabismologists have been devoted to reducing postoperative adhesions using a variety of materials, including polyglactin 910 mesh [1], mitomycin C [2, 3], and Seprafilm (Genzyme, Cambridge, MA) [4]. However, these methods have problems with their associated complications, unavailability, or inconsistent results. There is also a debate as to whether mitomycin C reduces inflammatory response after strabismus surgery significantly [2, 3].

Small guanosine triphosphatase (GTPase) Rho-kinase, a member of the Rho-kinase subfamily of the Ras superfamily of monomeric GTPases, constitutes an important modulator of vascular smooth muscle contraction [5–8]. Rho-kinase and its downstream effector are important mediators of not only vascular contraction but also actin cytoskeleton reorganization [9, 10], cellular morphology [11], motility [12], adhesion, and proliferation [5–8]. Due to its effects on various cellular functions, Rho-kinase has attracted significant interest as a potential target for the treatment of a wide range of pathological conditions including cancer, neuronal degeneration, kidney failure, asthma, glaucoma, osteoporosis, erectile dysfunction, and surgical adhesion [13–15]. Recent studies have shown that the Rho/Rho-kinase pathway is associated with tissue fibrosis and inflammation. It has also been demonstrated that the Rho/Rho-kinase pathway is involved in the tissue fibrosis process through regulation of TGF-*β* activation [16–18].

Therefore, we hypothesized that Y-27632 could be used as an adjuvant to suppress inflammation and fibrosis after strabismus surgery. The present study was conducted to investigate the effect of Y-27632 on postoperative inflammation and fibrosis following extraocular muscle surgery in a rabbit model.

## 2. Materials and Methods

### 2.1. Rabbits

In total, 16 eyes from 8 New Zealand white rabbits (each weighing 2.0–3.0 kg, 20 weeks old) were used for this study. All rabbits had anatomically normal eyes. All experiments were conducted in accordance with the tenets of the ARVO Statement for the Use of Animals in Ophthalmic and Vision Research. The protocol followed the guidelines of the Declaration of Helsinki and was approved by the Institutional Review Board of The Catholic University of Korea, College of Medicine.

### 2.2. Surgical Procedures

Each rabbit was anesthetized with intramuscular xylazine hydrochloride (5 mg/kg) and ketamine hydrochloride (40 mg/kg) and received a topical anesthetic containing 0.5% proparacaine hydrochloride (Alcaine 0.5%; Alcon, USA).

In both eyes of each rabbit, the superior conjunctiva and Tenon's capsule were opened, and the superior rectus muscle (SRM) was exposed using cotton swabs and a muscle hook. The SRM was then placed on a double-armed 6-0 Vicryl (polyglactin) suture that was located near the insertion site and detached from the globe. The SRM was sutured back to its original insertion after detachment, and the conjunctival suturing was performed with an interrupted 8-0 Vicryl suture.

In the right eye (treated eye) of each rabbit, 0.2 mL of 10 *μ*M Y-27632 was injected; 0.2 mL saline was injected into the left eye (control eye) subconjunctivally. To prevent infection, ofloxacin eye drops (Tarivid, Santen, Osaka, Japan) were applied once on each eye after surgery.

Rabbits were randomly selected for sacrifice using barbiturate anesthesia in a group of 4 (8 eyes) at postoperative day 3 and week 4. All eyes were enucleated and examined.

### 2.3. Evaluation of Inflammation

After animal sacrifice at postoperative day 3 and week 4, the sites of muscle reattachment were fixed in 10% formalin for 24 h, stored in 70% alcohol, and embedded in paraffin. Sequential 5 *μ*m sections of the wound site were prepared. Sections were examined microscopically using hematoxylin and eosin (H&E) staining and immunofluorescence. The sections were examined using rabbit polyclonal antibodies for Ki-67, CD11*β*+, and F4/80. The antibody-positive cell staining level was graded by a masked observer based on a histologic grading scale of 1 to 4 (1 = 0–10%, 2 = 10–30%, 3 = 30–50%, and 4 = >50% antibody-positive cell density).

### 2.4. Assessment of Late Adhesion and Fibrosis

Both eyes from 4 rabbits were examined regarding their gross adhesion score to evaluate postoperative fibrosis 4 weeks after the operation. Adhesion score severities were from 0 to 3 according to the criteria in a previous report, where 0 indicated no adhesion, 1 indicated adhesion easily separated with blunt dissection, 2 indicated mild-to-moderate adhesion with the freely dissectible plane, and 3 indicated moderate-to-dense adhesion with difficult dissection or a nondissectable plane [19].

Sections were also stained using Masson's trichrome staining kit (HT15, Sigma-Aldrich, St. Louis, MO, USA) and a fibronectin staining kit for quantification of collagen. Fibrosis was graded according to the amount of collagen as follows: 0, no fibrosis; 1, mild perimuscular fibrotic reaction (stained collagen was detectable only in thin bands immediately adjacent to muscle); 2, easily detected thick bands; 3, well-developed, dense bands of collagen; and 4, severe fibrotic response replacing large areas [20].

### 2.5. Statistical Analysis

All statistical analyses were performed using the SPSS software (ver. 17.0; SPSS, Chicago, IL, USA). Wilcoxon signed-rank tests were applied to make the comparison between the two postoperative groups. Values of *p* < 0.05 were considered statistically significant.

## 3. Results

During the follow-up period, all rabbits survived and remained in good health until sacrifice. No severe systemic complications were observed in any of the animals.

### 3.1. Acute Inflammation

Histologic examination by H&E staining showed inflammatory cell infiltration around the SRM and surrounding tissue in both groups.

**Figure 1:**
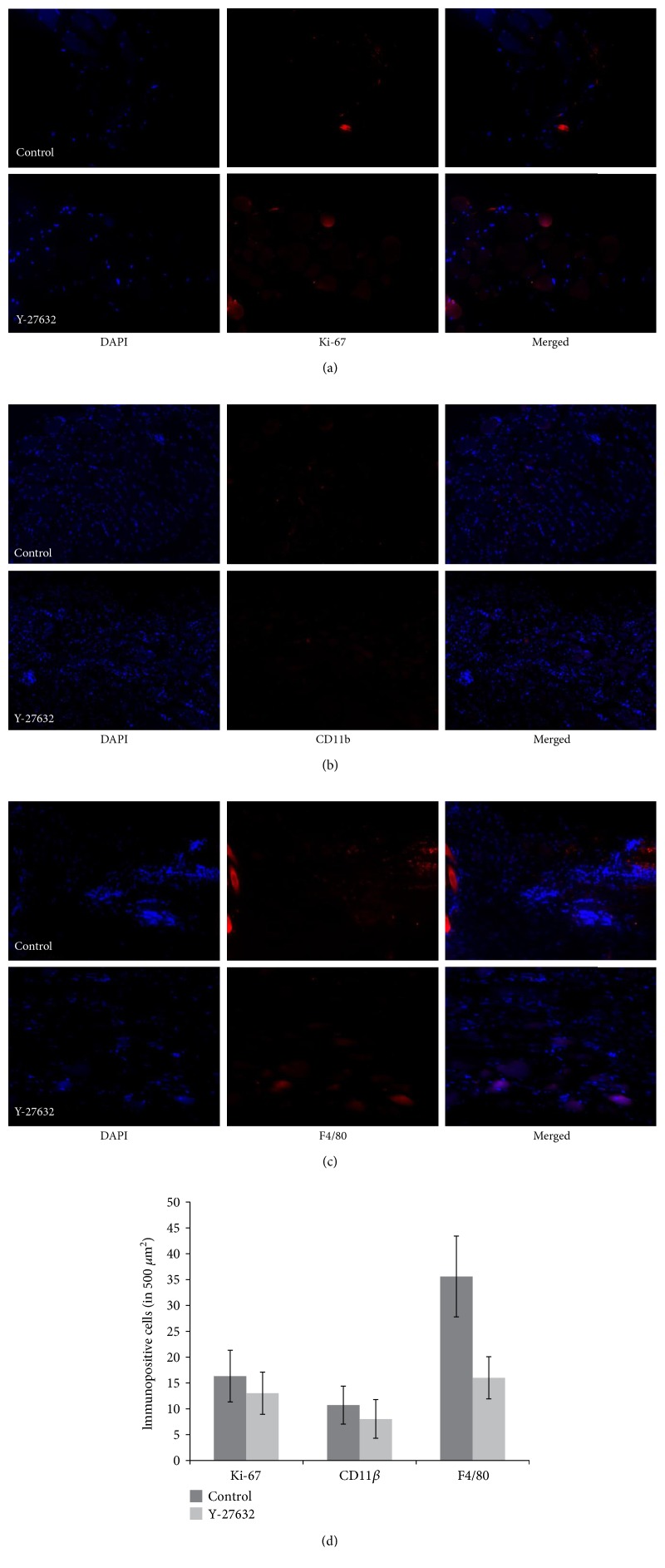
Immunohistochemical staining for Ki-67 (a), CD11 beta (b), and F4/80 (c) of the superior rectus muscle (SRM) 3 days after reinsertion of the SRM in a rabbit model: the normal saline injection group (above) and the Y-27632 injection group (below). Compared to the control group in which saline was injected into the SRM, only F4/80-positive areas were less present in the Y-27632 injection group (magnification, ×100). Immunepositive cells of Ki-67, CD11 beta, and F4/80 at 3 days after reinsertion of the SRM in a rabbit model (d).

Immunofluorescence of Ki-67 and CD11*β*+ was not significantly different in the Y-27632 injection group compared to the control at 3 days (*p* = 0.500, 0.054) and 4 weeks (*p* = 0.235, 0.692), respectively, postoperatively. However, F4/80 antibody expression was significantly different in the Y-27632 injection group at both 3 days and 4 weeks postoperatively (*p* = 0.038, 0.031) (Figures 1() and 2()).

### 3.2. Late Fibrosis

The SRM/conjunctiva adhesion score differed significantly between the groups (*p* = 0.034). However, the SRM/sclera adhesion score did not differ significantly between the groups (*p* = 0.178) 4 weeks after extraocular muscle surgery.

**Figure 3:**
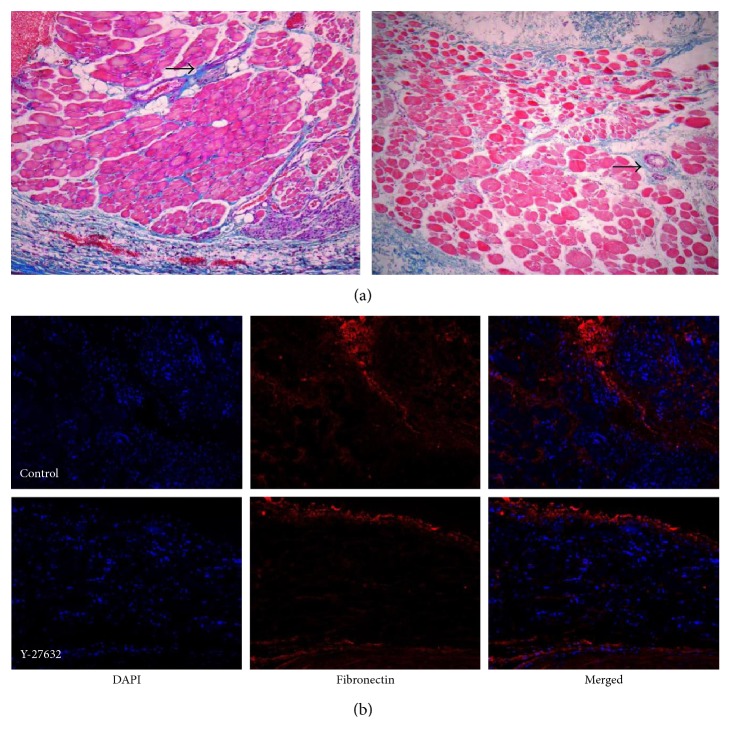
Light microscopy findings (Masson trichrome; original magnification, ×100) of the SRM 4 weeks after surgery (a): the normal saline injection group (left) and the Y-27632 injection group (right). Noted fibrosis within the muscle bundles was observed in the control group. Some muscle fiber was stained red in addition to the blue color (arrow). Mild fibrosis was observed in the Y-27632 group. Light microscopy findings (fibronectin; original magnification, ×100) of the SRM 4 weeks after surgery (b): the normal saline injection group (above) and the Y-27632 injection group (below).

H&E staining revealed some degree of granulation tissue with fibrosis around the SRM in both groups. Masson's trichrome stain in the SRM at postoperative 4 weeks presented some degree of blue-colored collagen in the endomysium in both groups. In addition, fibronectin stain in the SRM at postoperative 4 weeks presented some degree of red-colored tissues. The degrees of fibrosis in both stainings were lower in the Y-27632 injection group compared to the control, but not so significant (Figures 3(a) and 3(b)).

In addition, the muscle fibers were more highly separated by collagen deposition in the control group. With regard to postoperative changes, some muscle fibers were stained red faintly in the control group, whereas most were stained red distinctly in the Y-27632 injection group.

## 4. Discussion

In the present study, we demonstrated that the inhibitor of Rho-kinase, Y-27632, was able to reduce inflammation and SRM/conjunctiva adhesion in rabbit eyes following extraocular muscle surgery. Our results show that immunofluorescence staining of F4/80 decreased at postoperative day 3 and week 4. F4/80, a macrophage marker [21], exhibited lower positivity in the Y-27632 injection group compared to the control, implying that Y-27632 decreased the number of macrophages recruited to the wound site; that is, Y-27632 may suppress the macrophage-mediated inflammatory response. The protein serine/threonine kinase ROCK1 is the main effector of Rho GTPase RhoA, and the RhoA/ROCK pathway is the main regulator of actin cytoskeleton and actin-related functions in the cells. During the immune response, the movement of macrophages toward the target relies on proper organization of actin cytoskeleton and focal adhesions in the front and back of the cell. Y-27632 is a recently identified specific inhibitor of the ROCK-ROK family of protein kinases. Inhibition of ROCK1 by Y-27632 abolishes macrophage polarity and reduces their podosomes, motility, and phagocytosis and increases matrix degradation [22–25].

One of the predominant inflammatory cells within a wound is the macrophage at 48 to 96 h after wounding. Macrophages act as a major source of various cytokines and growth factors and are required to support cellular recruitment and activation, matrix synthesis, angiogenesis, and remodeling. Unlike neutrophils, macrophages remain within a wound until healing is complete [26].

Immunofluorescence staining of F4/80 was decreased in the Y-27632 injection group, but an expression of CD11*β* and Ki-67 at postoperative day 3 and week 4 was not different between the groups. CD11*β* is expressed on the surface of many leukocytes involved in the innate immune system, including monocytes, granulocytes, macrophages, and natural killer cells [27]. Additionally, Ki-67 is a cellular marker of proliferation [28] with which it is strictly associated. Our results imply that Y-27632 injection may decrease the inflammatory response mediated by macrophages, but this does not occur by either inhibition of leukocyte recruitment or inflammatory cell proliferation. Our study shows only the results of postoperative day 3 and week 4. Therefore, further studies are needed to demonstrate the intermediate process in order to confirm that the results of three days last up to four weeks.

Macrophages and fibroblasts release numerous growth factors and cytokines that contribute to fibroblast migration: fibroblast growth factor (FGF), IGF-1, vascular endothelial growth factor (VEGF), IL-1, IL-2, IL-8, platelet-derived growth factor (PDGF), TGF-*α*, TGF-*β*, and TNF-*α*. Fibroblasts are activated to proliferate and begin synthesizing collagen. These activated fibroblasts are related to adhesion and fibrosis [26].

In this study, the SRM/conjunctiva adhesion score differed between the groups; this might be interpreted as a reduction in the macrophage-mediated inflammatory response, thus reducing adhesion and fibrosis. However, the SRM/sclera adhesion score and Masson's trichrome/fibronectin staining did not differ significantly. Therefore, we believe that other factors in addition to macrophages might be involved in the inflammatory process and the activation of fibroblasts in the extraocular muscle and surrounding connective tissue. Analysis of the tissue should consider the possibility of biases due to lengthened scars, consisting of amorphous connective tissue. Therefore, all procedures of this study were conducted by a single ophthalmologist who had experiences of strabismus operation more than ten years. In addition, factors that distinguish stretched scars from classic slipped muscles included minimal or no limitation of versions, less separation of the tendons from the sclera, and thicker appearance of the scar segments [29].

Rho-GTPase participates in signaling pathways leading to the formation of actin stress fibers and focal adhesions. Rho is also involved in diverse physiological functions associated with cytoskeletal rearrangements, such as cell morphology, cell motility, cytokinetics, and smooth muscle contraction. Rho-associated protein kinases (ROCKs) play a key role in focal adhesions and stress fiber formation in cultured fibroblasts and epithelial cells and in Ca^+2^ sensitization of smooth muscle cells [30]. Y-27632 is a recently identified specific inhibitor of the ROCK-ROK family of protein kinases. Y-27632 inhibits Ca^+2^-sensitive smooth muscle contraction and has been suggested as a useful therapeutic for hypertension [13].

Recent studies have shown that the Rho/Rho-kinase pathway is associated with tissue fibrosis and inflammation [31–34]. The Rho/ROCK-mediated pathway plays a role in the infiltration of inflammatory cells both *in vitro* and *in vivo* [16, 17]. Y-27632 also prevented the upregulation of *α*-smooth muscle actin (*α*-SMA), a marker of tissue fibrosis, and inhibited tubulointerstitial fibrosis in mouse kidney with unilateral ureteral obstruction [14]. Another study reported that the selective ROCK inhibitor, fasudil, had beneficial effects on bleomycin-induced pulmonary fibrosis in mice. The number of infiltrated inflammatory cells in bronchoalveolar lavage fluid (BALF) was attenuated by fasudil [32]. The present results suggest that Y-27632 inhibits the macrophage-mediated inflammatory response and reduces adhesion and fibrosis.

In conclusion, our data suggest that injection of Y-27632 inhibited inflammation and adhesion during the wound healing process. Y-27632 may thus have significant potential as prophylaxis for postoperative adhesion syndrome in extraocular muscle surgery.

## Figures and Tables

**Figure 2:**
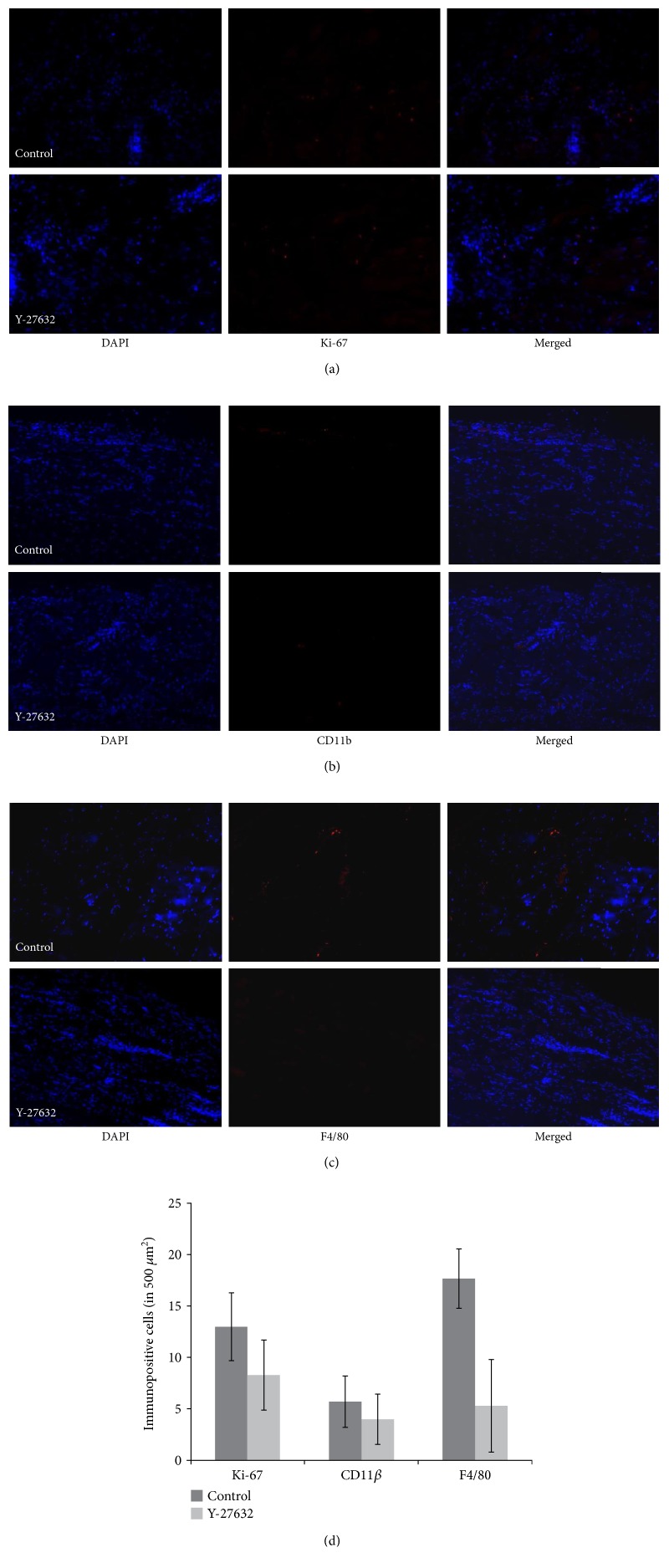
Immunohistochemical staining for Ki-67 (a), CD11 beta (b), and F4/80 (c) of the SRM 4 weeks after reinsertion of the SRM in a rabbit model: the normal saline injection group (above) and the Y-27632 injection group (below). Compared to the control group in which saline was injected into the SRM, only F4/80-positive areas were less present in the Y-27632 injection group (magnification, ×100). Immunepositive cells of Ki-67, CD11 beta, and F4/80 at 4 weeks after reinsertion of the SRM in a rabbit model (d).
